# Thrombotic Thrombocytopenic Purpura Associated with Myelodysplastic Syndrome

**DOI:** 10.7759/cureus.7364

**Published:** 2020-03-22

**Authors:** Zohra R Malik, Nassim Mokraoui, Zareen Razaq, Gabriela L Severiano, Arnaud Yanogo

**Affiliations:** 1 Internal Medicine, St. John's Episcopal Hospital, Far Rockaway, USA; 2 Internal Medicine, Ghurki Trust Teaching Hospital, Lahore, PAK; 3 Internal Medicine, Ross University School of Medicine, New York, USA; 4 Internal Medecine, St John's Episcopal Hospital, Far Rockaway, USA

**Keywords:** myelodysplastic syndrome, adamts13, ttp

## Abstract

Myelodysplasia and thrombotic thrombocytopenic purpura (TTP) are both rare diseases. TTP is a blood abnormality in which blood clots form in blood vessels leading to fatal outcomes. Myelodysplastic syndrome is a group of disorders caused by poorly formed blood cells or ones that do not work properly. We are hereby presenting the case of a 69-year-old female who presented with anemia, thrombocytopenia, changes in mental status and reduced kidney function, and further investigations revealed that the patient had underlying myelodysplasia.

## Introduction

Thrombotic thrombocytopenic purpura (TTP) is a thrombotic microangiopathy caused by severely reduced activity of the von Willebrand factor-cleaving protease ADAMTS13. It is characterized by small-vessel, platelet-rich thrombi that cause thrombocytopenia and microangiopathic hemolytic anemia. Some patients have neurological abnormalities, mild renal insufficiency and low-grade fever. Most cases of TTP are acquired, caused by autoantibody inhibition of ADAMTS13 activity. Hereditary TTP, caused by ADAMTS13 gene mutations, is much less common [1]. Myelodysplastic syndrome (MDS) is characterized by multilineage cytopenias due to ineffective hematopoiesis, and predominantly affects the elderly with a peak incidence between the ages of 60 and 75 years.

## Case presentation

**Figure 1:**
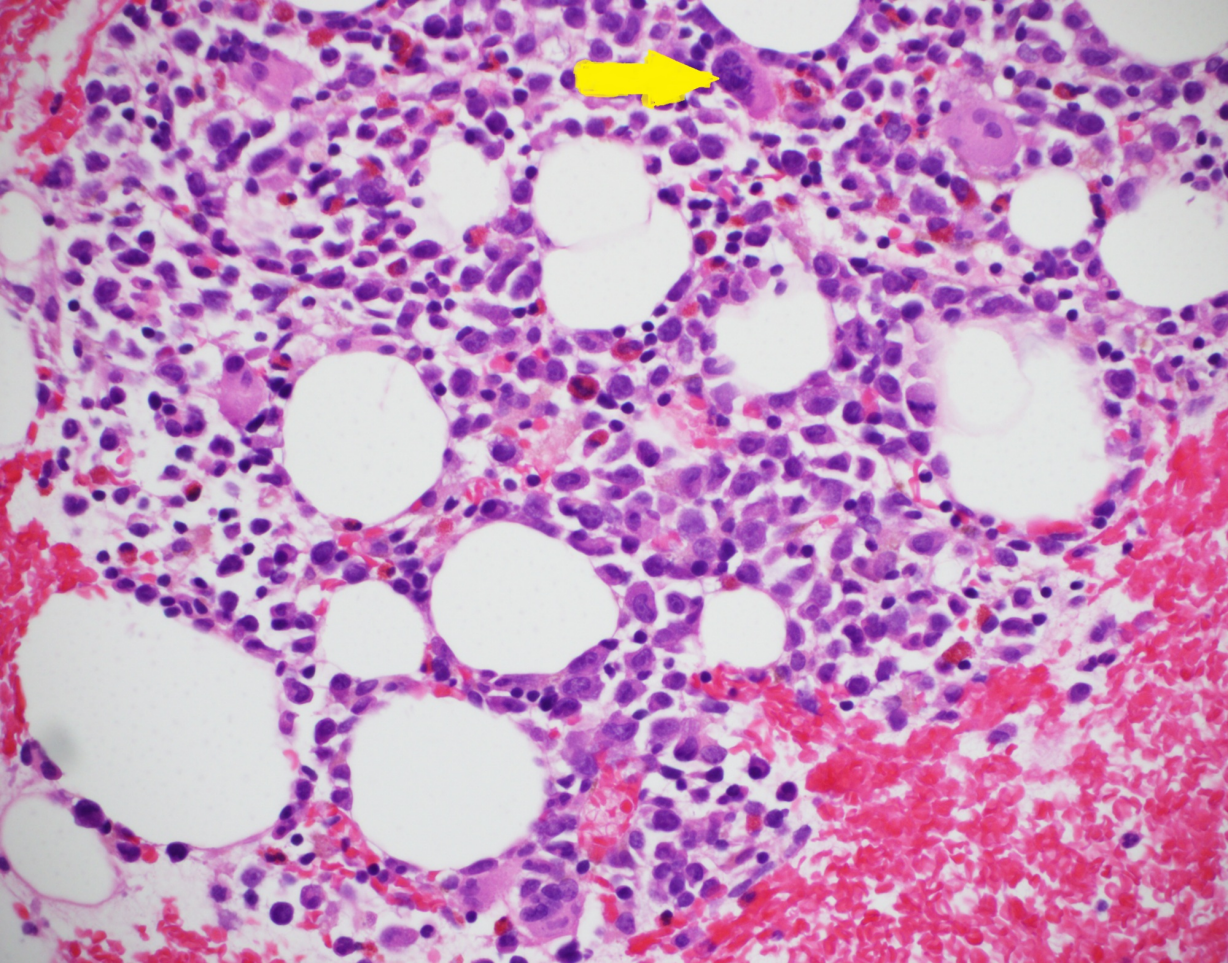
High-power view of bone marrow showing a megakaryocyte (yellow arrow) and erythyroid hypoplasia

**Figure 2:**
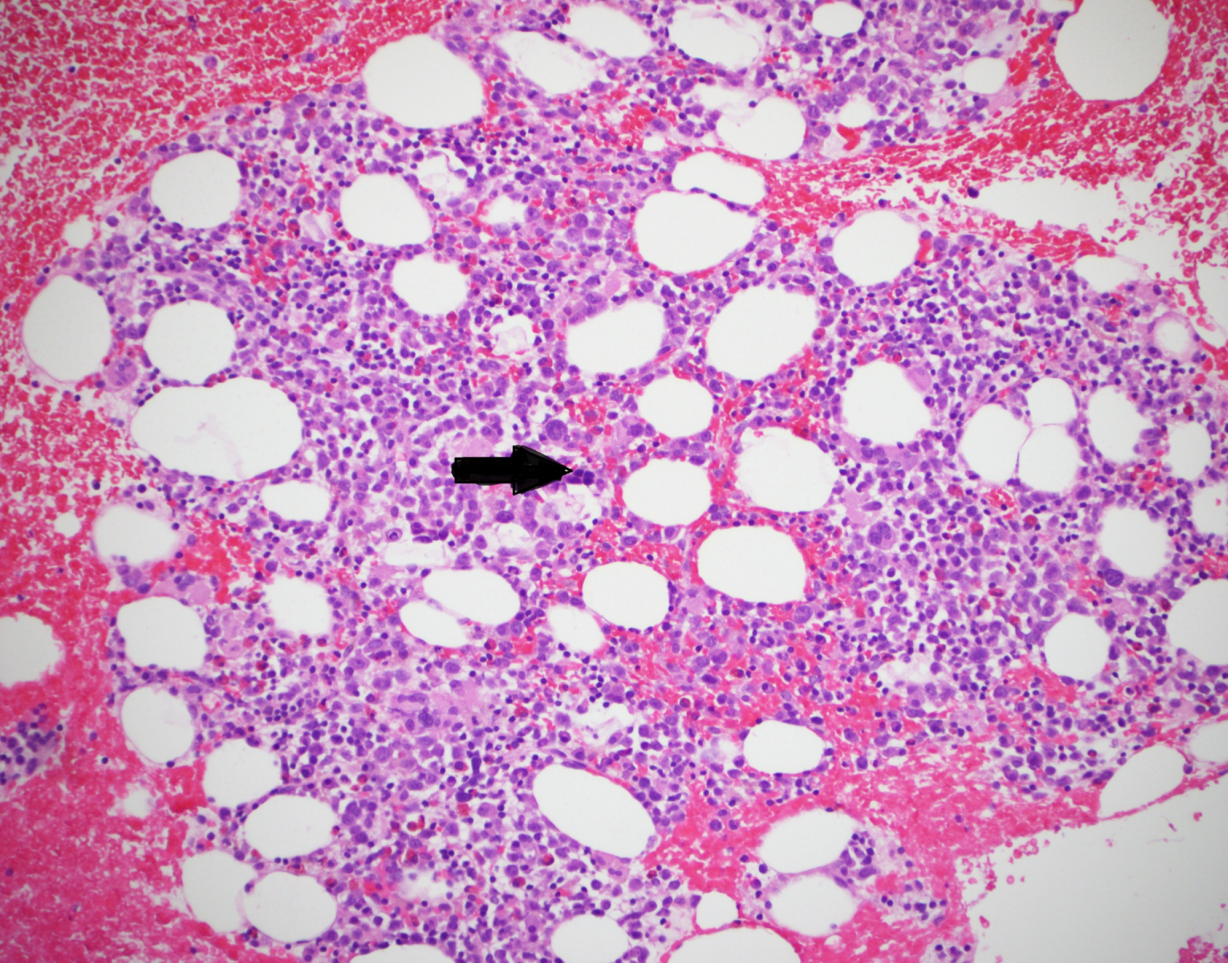
Low-power view of bone marrow showing hypocellularity and erythyroid hypoplasia (black arrow showing an occasional erythyrocyte)

A 69-year-old female with diabetes mellitus type 2, hypertension and coronary artery disease was admitted with complaints of fever, altered consciousness and hypotension. She had been chronically anemic refractory to blood transfusions. Lab work-up showed hemoglobin concentration 6.3 g/dL (normal 12.0-18.0 g/dL), hematocrit 21.2% (normal 37%-47%), red blood cells 2.62x10^6 ^cells/µL (normal 4.20-5.40x10^6^ cells/µL), platelet count 41,000 cells/µL (normal 130,000-400,000 cells/µL), lactate dehydrogenase 635 U/L (normal 313-618 U/L), total bilirubin 1.6 mg/dL (normal 0.2-1.3 mg/dL), prothrombin time 14.2 seconds (normal 10.0-12.9 seconds), activated partial thromboplastin time 67.5 seconds (normal 27.5-36.3 seconds), blood urea nitrogen 34.0 mg/dL (normal 7.0-17.0 mg/dL) and creatinine 2.06 mg/dL (normal 0.52-1.04 mg/dL). ADAMTS13 activity was found to be less than 10%, which is defined as severe deficiency of ADAMTS13. Blood cultures and urine cultures were negative, ruling out any infectious etiology of presentation. A diagnosis of TTP was made owing to the presence of altered state of consciousness, renal failure, anemia and thrombocytopenia. A bone marrow biopsy was done to find out cause of anemia. The biopsy raised the possibility of MDS. Figure 1 shows a high-power view of bone marrow with megakaryocyte (yellow arrow). Figure 2 shows a low-power view of hypocellular bone marrow and erythroid hypoplasia (black arrow showing an occasional erythyrocyte).

## Discussion

The diagnosis of TTP in our case was made based on clinical and laboratory findings of hemolytic anemia, thrombocytopenia, neurological symptoms and renal failure. Although TTP usually occurs without other underlying diseases, some cases have been reported in association with a variety of conditions such as pregnancy, infections, toxins and autoimmune disorders [2]. In order to diagnose TTP in the acute phase of the disease, it is not essential to assay ADAMTS13 [3]. After having ruled out other thrombotic microangiopathies, patients can still be appropriately diagnosed with TTP without the ADAMTS13 assay. There is no effective therapy for TTP, but plasma therapy (plasma exchange, plasmapheresis or infusion), alone or combined with other forms of therapy, can dramatically improve the prognosis of patients with TTP. The mechanism by which the therapy works is not well understood [4]. The decision to implement plasma therapy (infusion in patients with an inherited disease, exchange in acquired disease) does not warrant the availability of ADAMTS13 values in real time [3]. Other forms of therapy are corticosteroids, antiplatelet agents, high doses of immunoglobulin and vincristine [5-8]. A splenectomy is a treatment option of last resort [9]. Early diagnosis and prompt treatment with plasmapheresis may improve the outcome of TTP patients with myelodysplasias [4]. Sepsis was low on the differential for causing this presentation. Blood cultures, urine cultures and sputum cultures were all negative. Clostridium difficile was negative. Heparin-induced thrombocytopenia panel was negative. The patient had a massive leukocytosis which could be an acute progression to acute myeloid dysplasia. Physical examination showed diffuse purpura of bilateral upper and lower extremities and the abdomen. The patient did not undergo plasmapheresis for suspected TTP and did not receive treatment for suspected MDS as she was a poor candidate due to age and high mortality risk. The patient’s IPSS (International Prognostic Scoring System) for MDS was intermediate to high risk. Physicians should keep in mind that TTP occasionally arises as a serious complication of myelodysplasia and anyone presenting with signs and symptoms suggestive of TTP should be further investigated, as prompt treatment is the key to survival.

## Conclusions

The possibility of an association between TTP and MDS should always be considered especially in a person presenting with altered levels of consciousness, renal function abnormalities, anemia, thrombocytopenia and biopsy showing myelodysplastic cells. 
